# The role of Zearalenone in epigenetic modifications of candidate genes in Nellore heifers

**DOI:** 10.1007/s11250-026-04978-y

**Published:** 2026-04-11

**Authors:** Lethissia Amorim da Silva Coelho, Rondineli Pavezzi Barbero, Pedro Ruiz Martins Tapajos Pereira, Carolina Emiliano Bastos Polido, Ana Clara Souza Resende de Aguiar, Huarrisson Azevedo Santos, Marco Roberto Bourg de Mello, Elisandra Lurdes Kern, Denise Carleto Andia, Marina Mortati Dias Barbero

**Affiliations:** 1 Instituto de Zootecnia, Universidade Federal Rural do Rio de Janeiro, Seropédica, RJ Brazil; 2 Instituto de Veterinária, Universidade Federal Rural do Rio de Janeiro, Seropédica, RJ Brazil; 3 Instituto de Ciências Biológicas e da Saúde, Universidade Federal Rural do Rio de Janeiro, Seropédica, RJ Brazil; 4 Insituto de Ciências da Saúde, Faculdade de Odontologia, Universidade Paulista, São Paulo, SP Brazil

**Keywords:** CpG island, *CYP1A1*, *CYP21A2*, *CYP1B1*

## Abstract

Mycotoxins are toxic secondary metabolites produced by fungi that proliferate in stored feeds such as grains and silage. Zearalenone (ZEN) is a frequently detected mycotoxin with well-known estrogenic activity. This study aimed to evaluate the effects of ingesting ZEN-contaminated feed on the methylation and hydroxymethylation of CpG islands located in the candidate genes *CYP1A1*, *CYP21A2*, and *CYP1B1* in Nellore heifers. Twenty heifers were confined for 12 weeks during the finishing phase and assigned to two groups: a control group (non-contaminated feed) and a ZEN-treated group (approximately 300 ppb). After slaughter, liver samples were collected, and genomic DNA was extracted. DNA was treated with T4-β-glucosyltransferase and digested with the restriction enzymes *MspI* and *HpaII*. CpG island methylation and hydroxymethylation were quantified by real-time PCR. Statistical analyses were performed using analysis of variance followed by *t*-tests, and *p*-values were adjusted for multiple comparisons using the false discovery rate (FDR). No significant differences were observed in methylation or hydroxymethylation levels of *CYP1A1* between groups after FDR adjustment. In contrast, animals exposed to ZEN-contaminated feed exhibited increased DNA methylation in the CpG island of *CYP1B1*, and this effect remained statistically significant after FDR correction. For *CYP21A2*, differences in hydroxymethylation observed in the unadjusted analysis were attenuated after FDR adjustment and are therefore interpreted as a trend. These findings indicate that ZEN exposure induces gene-specific epigenetic modulation in the liver of Nellore heifers, with a robust effect observed for *CYP1B1*. The results highlight the importance of monitoring ZEN contamination in cattle feed and support the need for further studies integrating epigenetic, transcriptomic, and endocrine analyses to clarify the biological implications of mycotoxin exposure in beef cattle.

## Introduction

Mycotoxins are secondary metabolites that are naturally produced by fungi, usually as a defense mechanism against these microorganisms as they mature (Kemboi et al. 2020). Food contamination by mycotoxins can occur both in field conditions and during storage, and is influenced by intrinsic and extrinsic factors such as the environment, climatic conditions, and fungal species involved (Hussein and Brasel 2001). This contamination can compromise the quality of plants and consequently affect the animals that consume them (Freire et al. 2007). Toxic effects range from skin lesions to more severe manifestations, such as hepatotoxicity, nephrotoxicity, neurotoxicity, hematotoxicity, genotoxicity, and, in extreme cases, death. Acute and chronic toxicities of mycotoxins have been extensively studied, highlighting the various toxicological pathways involved. Therefore, understanding the toxicity mechanisms induced by mycotoxins is essential for implementing preventive measures and protecting human and animal health.

Increasing demand for food has driven the intensification of livestock production, including beef cattle farming. To optimize weight gain and productivity, grains and silage are widely used in cattle feeding as they contribute to reducing the production cycle of animals (Barbero et al. 2021). However, these feedstuffs present a high risk for mycotoxin contamination (Custódio et al. 2019). In ruminants, ingested Zearalenone (ZEN) is converted into α-zearalenol and β-zearalenol by ruminal microorganisms (Kemboi et al. 2020). α-zearalenol has a higher estrogenic potency than ZEN because it can be converted into zeranol, a compound with anabolic effects. However, its toxic impact is reduced owing to lower absorption, and when absorbed, it is converted into β-zearalenol in the liver (Kuiper-Goodman et al. 1987; Rai et al. 2020). β-Zearalenol, in turn, exerts toxic effects on endometrial cells, although its affinity for estrogen receptors is low (Bottalico et al. 1985). Although ruminants are less sensitive to ZEN exposure than monogastric animals due to the metabolism of this toxin in the rumen (Fink-Gremmels 2008; Liu and Applegate 2020), its elimination can be compromised in cases of ruminal acidosis, leading to increased ZEN bioavailability and potential toxic effects (Takagi et al. 2011). In studies with pigs, Doll et al. (2004) detected ZEN and its metabolites in both bile and liver, demonstrating the persistence of the toxin in the organism.

As reviewed by Zhang (2015), the effects of diet on epigenetic mechanisms, including DNA methylation patterns, have been widely studied. DNA methylation involves the addition of a methyl group to cytosine (C), located within a CpG island in the gene promoter region (Zhang 2015). The study of methylation patterns is justified by their impact on the regulation of gene expression.

Evidence from nutrigenomic studies indicates that diet can influence DNA methylation processes, thereby modulating gene expression (Maugeri and Barchitta 2020). The degradation of mycotoxins primarily occurs in the liver, and oxidation is one of the key steps in this process (Tolosa et al. 2021). Some enzymes involved in mycotoxin degradation belong to the cytochrome P450 gene superfamily (Berenbaum et al. 2021). If adverse effects occur in the reproductive system of animals, they may be linked to the failure of ZEN degradation and the consequent methylation of the genes responsible for this process.

When the mycotoxin ZEN is ingested, part of its detoxification process occurs in the liver, where cytochrome P450 family enzymes play a crucial role. In studies on mice, for instance, genes from this family showed altered expression in the liver following exposure to ZEN (Duca et al. 2010). In pigs, Gajecka et al. (2017) demonstrated that exposure to low doses of ZEN affects multiple biological targets within an organism. These authors reported that ZEN-induced hyperestrogenism results in changes in the mRNA activity of selected enzymes, including those in the cytochrome P450 family, affecting the regulation of host steroid hormones, bacterial gene virulence, and detoxification processes. Additionally, they observed reduced intestinal activity, leading to energy deficits.

This study hypothesized that ZEN ingestion by Nellore heifers may alter the methylation patterns of genes involved in the degradation of this mycotoxin, specifically those encoding proteins of the cytochrome P450 family, including *CYP1A1*, *CYP21A2*, and *CYP1B1*. Understanding the gene regulation mechanisms in response to ZEN intoxication may contribute to a better understanding of detoxification processes in cattle and aid in developing strategies to mitigate the effects of this mycotoxin.

## Materials and methods

### Animals, treatments, and sample collection

Twenty non-pregnant, healthy Nellore heifers (*Bos taurus indicus*) aged ≥ 18 months with an initial average body weight of 330 ± 30 kg were used in this study. The animals were divided into two groups: 10 heifers in the control group and 10 that consumed ZEN-contaminated feed (concentrate). At the beginning of the experiment, all animals were weighed and fed a diet composed of 70% forage and 30% concentrate (on a dry matter basis) to evaluate two treatments: (1) control (non-contaminated feed) and (2) ZEN-contaminated feed (± 300 ppb). This dose is considerably lower than the lethal dose for cattle (Chang et al. 2017) and is commonly found in feedlot diets (Custódio et al. 2019). The feeding protocol was described by Pião et al. (2023). After 84 days of confinement, the animals were slaughtered in a commercial slaughterhouse, and liver samples were collected. The study was approved by the Ethics Committee on Animal Use of the IZ/UFRRJ (Approval No. 0028-10-2018).

### DNA extraction and quantification

DNA was extracted from the liver samples using the DNeasy Blood & Tissue Kit (QIAGEN), following the manufacturer’s specifications. DNA integrity was assessed by electrophoresis on 1% agarose gel. DNA was quantified using the Qubit™ fluorometer (Invitrogen™) and the Qubit dsDNA BR Assay Kit. The quality of the extracted DNA was analyzed using a NanoDrop 2000 spectrophotometer. Subsequently, the DNA was stored at -20 °C until further analysis.

### Candidate genes, CpG island identification and primer design

The selection of candidate genes was based on the degradative activity attributed to the cytochrome P450 (CYP) gene family (Berenbaum et al. 2021), with particular focus on *CYP1A1*, *CYP21A2*, and *CYP1B1*, as consistently reported in the literature (Frizzell et al. 2013; Kalayou et al. 2014; Mróz et al. 2022; Zepnik et al. 2001).

CpG islands were identified using the Genome Browser (https://genome.ucsc.edu/). Islands located near or within the candidate gene regions were selected. Based on the CpG island DNA sequence, a pair of primers was designed (Table 1) using Primer3Plus software (Untergasser et al. 2012) with *Bos taurus* ARS-UCD 2.0 as the reference genome. Primer quality was verified using the Beacon Designer-Free Edition tool (http://www.premierbiosoft.com/).

**Table 1:** Location of CpG islands and primers designed for each candidate gene

Candidate Gene	CpG Island Location	Primer Sequence
*CYP1A1*	21: 33951871.33952098	F: CTATGGGGACGTGCTGCA
		R: AGAGTCTGGGTTGAAGGTCA
*CYP21A2*	23: 27327147.27327896	F: TGCTCCCGAGTCACTTACAA
		R: ATAACGACCATGCCCTCAGG
*CYP1B1*	11: 20473998.20477669	F: CTTGTGATGCGTGGGTTCTC
		R: CGCCTCTCTATGACCCGATT

### Quantitative PCR assay for DNA methylation and hydroxymethylation

**Fig. 1:**
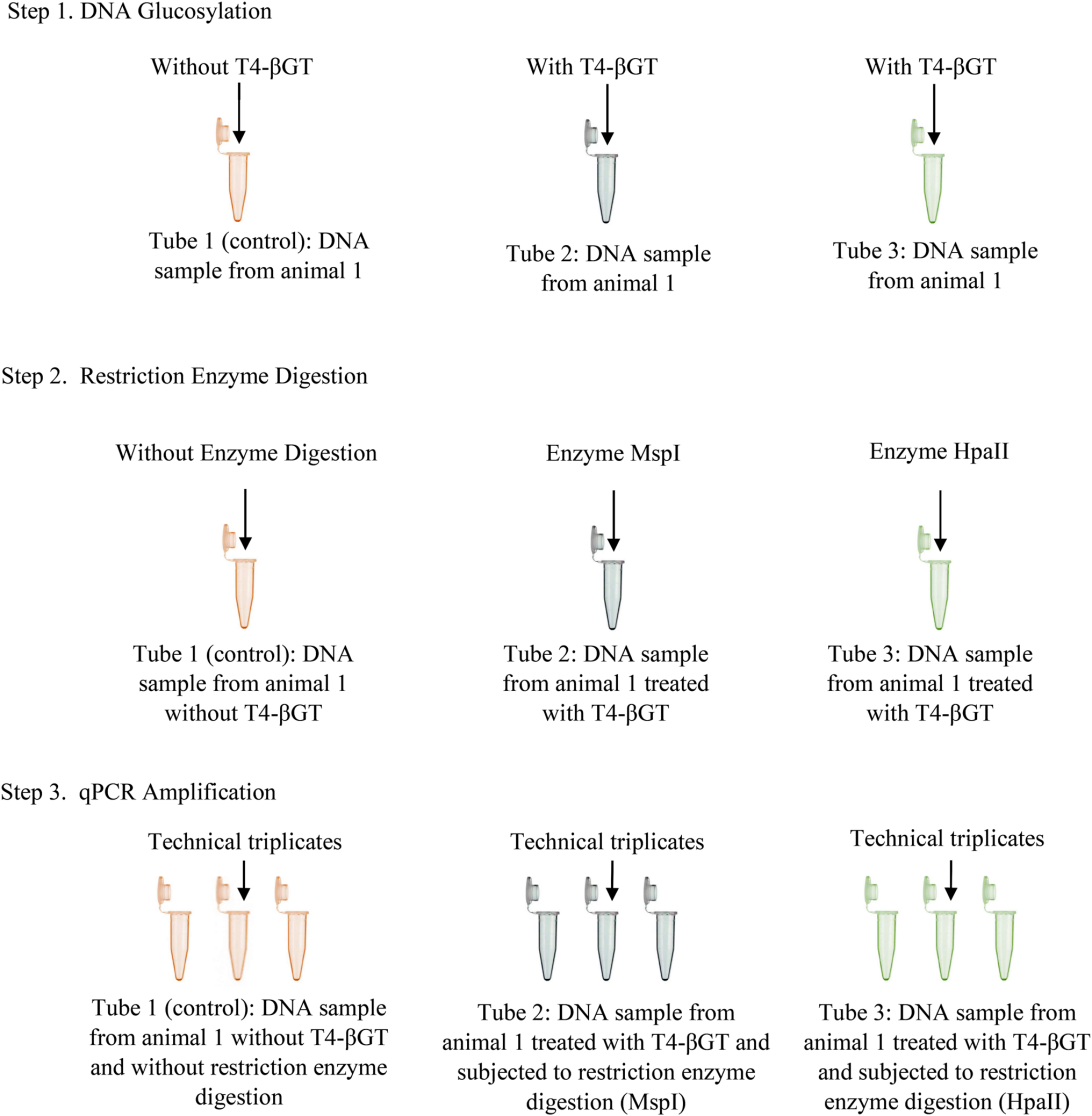
Workflow of DNA glucosylation, restriction enzyme digestion, and qPCR amplification used to estimate DNA methylation and hydroxymethylation levels. Each DNA sample was analyzed under three experimental conditions

Genomic DNA was initially treated with T4-β-glucosyltransferase (T4-BGT) (Thermo Scientific™), an enzyme that adds a glucose moiety to 5-hmC, allowing for differentiation between DNA methylation and hydroxymethylation. Each sample was analyzed under three experimental conditions (Fig. 1): (1) without glucosylation and without restriction enzyme treatment (control), (2) glucosylated and treated with the restriction enzyme *MspI*, and (3) glucosylated and treated only with the restriction enzyme *HpaII*.

Samples subjected to glucosylation (two tubes per DNA sample) were prepared with the following components: 0.4 µg of DNA, 2 µL of 10X Epi Buffer 4, 2 µL of 10X UDP-glucose, 0.4 µL of T4-BGT, and 7.6 µL of nuclease-free water, for a final volume of 20 µL. Non-glucosylated samples were prepared as follows: 0.4 µg of DNA, 2 µL of 10X Epi Buffer 4 (2 µL 10X UDP-glucose, and nuclease-free water (8 µL), resulting in a final volume of 20 µL. All the samples were incubated at 37 °C for 15 min, followed by enzyme inactivation at 65 °C for 20 min.

Next, *MspI* (Invitrogen™, Thermo Scientific™), *HpaII* (Invitrogen™ by Thermo Scientific™), or water (for the control) was added to the respective tubes, bringing the final volume to 25 µL, followed by incubation at 37 °C for 1 h. The samples treated with *HpaII* were incubated at 65 °C for 10 min to inactivate the enzyme.

Quantification of DNA methylation and hydroxymethylation was performed using a methylation-sensitive restriction enzyme–based qPCR approach, in which amplification reflects the presence or absence of enzyme cleavage rather than transcript abundance. In this context, classical qPCR quality controls applied to gene expression analyses, such as primer efficiency derived from serial dilutions and single-peak melting curves, are not mandatory, as discussed by Kurdyukov and Bullock (2016).

Real-time quantitative PCR (qPCR) was performed using each primer pair. The samples were amplified for 40 cycles using the StepOnePlus Real-Time PCR System (Applied Biosystems). The reaction mix was performed in a final volume of 10 µL containing 50ng/µL of DNA, 40 µM of gene-specific primer pair, 5 µL of Fast SYBR^®^ Green Master Mix 2x (Applied Biosystems), and 3 µL of nuclease-free water. The temperature and cycle duration followed the instructions for Fast SYBR ^®^ Green Master Mix. The melting curve was obtained in the temperature range of 60 °C to 95 °C, increments of 0.3 °C per cycle, a stabilization time of 30 s per increment, and continuous fluorescence acquisition in the SYBR™ Green channel.

Data analysis was conducted using the comparative Cq (cycle quantification) method, with sample normalization based on the control reaction (treated only with T4-BGT, without enzyme digestion) as the calibrator (Assis et al. 2018).

The Cq values obtained by qPCR were used to estimate the levels of methylation (M) and hydroxymethylation (H), as described by Assis et al. (2018), using the following calculations:

$$M=\frac{Hpa\:II-Msp\:I}{\mathrm{c}\mathrm{a}\mathrm{l}\mathrm{i}\mathrm{b}\mathrm{r}\mathrm{a}\mathrm{t}\mathrm{o}\mathrm{r}}$$

$$H=\frac{Msp\:I}{\mathrm{c}\mathrm{a}\mathrm{l}\mathrm{i}\mathrm{b}\mathrm{r}\mathrm{a}\mathrm{t}\mathrm{o}\mathrm{r}}$$

Where *HpaII* represents the Cq values obtained from samples treated with the *HpaII* restriction enzyme, *MspI* represents the Cq values obtained from samples treated with the *MspI* restriction enzyme, and calibrator refers to the samples that were not subjected to any enzymatic treatment.

### Statistical analysis

Statistical analyses were conducted using datasets in which animals were identified according to their experimental group (control or ZEN-treated). Data were subjected to analysis of variance (ANOVA, α < 0.05\alpha < 0.05α < 0.05), followed by t-test comparisons using the *agricolae* package (Mendiburu 2021) in R software (R Core Team 2026). To control for multiple testing across genes and epigenetic variables, p-values were adjusted using the false discovery rate (FDR) according to the Benjamini–Hochberg procedure (Benjamini and Hochberg 1995). Adjusted p-values (p_adj) < 0.05 were considered statistically significant.

## Results

The CpG islands analyzed were located in the following genomic regions (Table 1): for *CYP1A1*, within the gene region, specifically exon 2, corresponding to the first portion of the coding sequence; for *CYP1B1*, within the gene region, encompassing exons 2 and 3 as well as a downstream segment; and for *CYP21A2*, within the gene region, spanning exons 6 to 8.

**Fig. 2:**
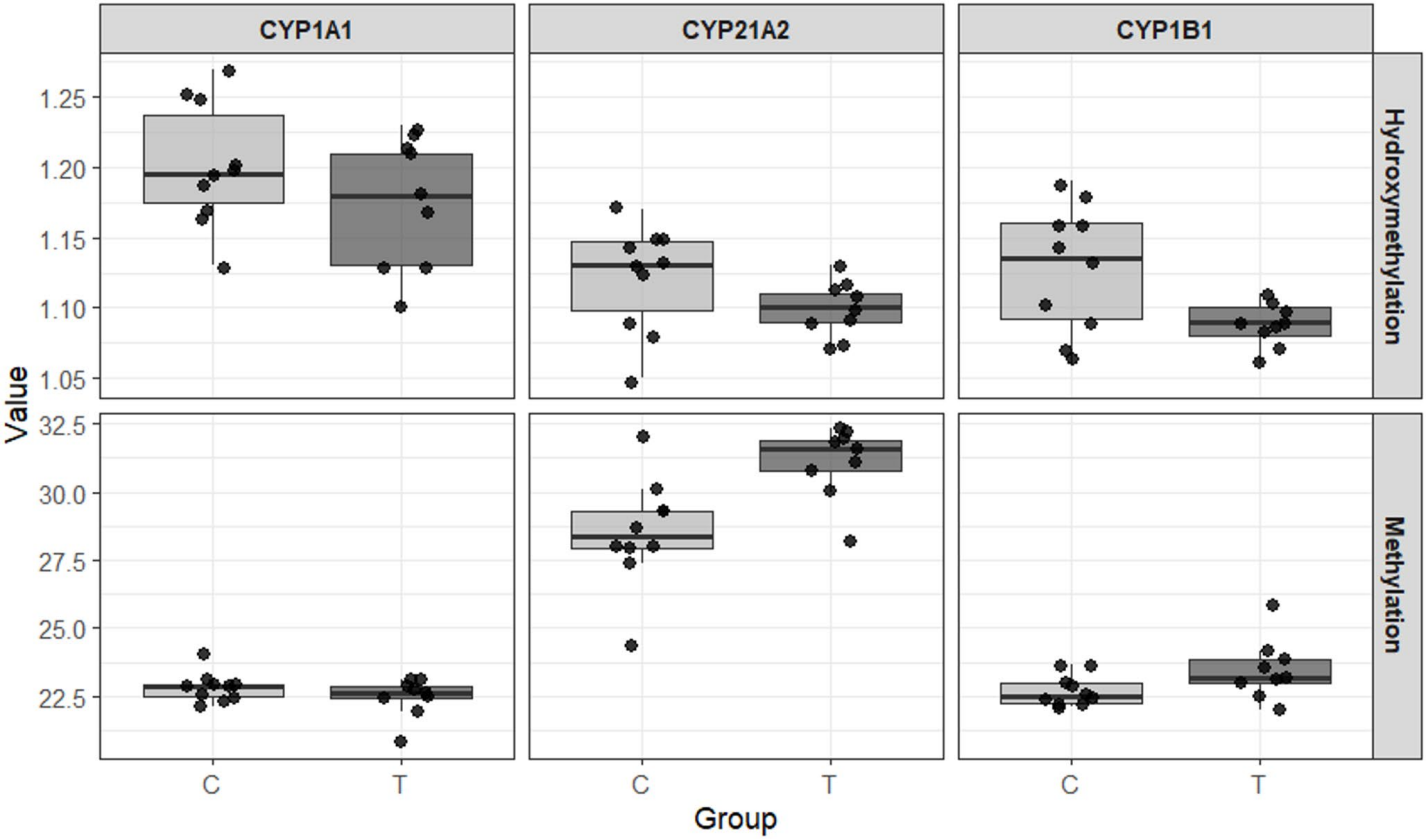
Comparison of DNA methylation and hydroxymethylation levels in CpG island regions of *CYP1A1*, *CYP21A2*, and *CYP1B1* between control (C) and ZEN-treated (T) Nellore heifers. Boxes represent interquartile ranges, horizontal lines indicate medians, and points represent individual animals. Control group: *n* = 10; ZEN-treated group: *n* = 9. One sample from the ZEN-treated group was excluded due to sample loss

The quantity and quality of the extracted DNA were suitable for analysis, except for one sample from the treatment group, which was excluded from the statistical analysis. The CpG island regions of the candidate genes *CYP1A1*, *CYP21A2*, and *CYP1B1* were successfully amplified using qPCR. Statistical analyses were performed based on estimated methylation and hydroxymethylation values (Tables 2 and 3). The Fig. 2 provides a visual comparison of DNA methylation and hydroxymethylation levels between control and ZEN-treated groups, highlighting the gene-specific epigenetic responses.

To control for multiple comparisons across genes and epigenetic variables, *p*-values were adjusted using the false discovery rate (FDR) according to the Benjamini–Hochberg procedure (Benjamini and Hochberg 1995). Adjusted *p*-values (FDR) are reported in Tables 2 and 3 and were used to determine statistical significance.

No significant differences were observed in the CpG island region of *CYP1A1* for either methylation or hydroxymethylation after FDR adjustment, indicating that consumption of ZEN-contaminated feed did not induce detectable epigenetic changes in this gene under the experimental conditions evaluated.

For *CYP21A2*, a difference in hydroxymethylation was observed between the control and treatment groups in the unadjusted analysis; however, this effect did not remain statistically significant after FDR correction and is therefore interpreted as a trend. Methylation levels in this gene did not differ significantly between groups after adjustment.

In contrast, *CYP1B1* showed a significant increase in DNA methylation in animals exposed to ZEN-contaminated feed compared with the control group, and this effect remained statistically significant after FDR correction. No significant differences were detected in hydroxymethylation levels for this gene.

**Table 2:** Statistical analysis using the t-test for the comparison of transformed methylation values between animals in the control and treatment groups

Gene	Animals	M	STD	Se	Min	Max	D	*p*-value	*p*-adj (FDR)
*CYP1A1*	C (10)	22.82	0.5274	0.1976	22.14	24.03	0.3605	0.2263	0.2371
	T(9)	22.46	0.7192	0.2083	20.81	23.14			
*CYP21A2*	C(10)	22.69	0.5700	0.2707	22.10	23.64	-0.7755	0.0652	0.1304
	T(9)	23.46	1.0900	0.2854	22.00	25.80			
*CYP1B1*	C(10)	28.51	1.9722	0.5354	24.40	32.00	-2.5900	0.0040	0.0240
	T(9)	31.10	1.3105	0.5644	28.20	32.30			

where C (10) = control group (non-contaminated feed; *n* = 10 animals); T (9) = ZEN-treated group (*n* = 9 animals), H = estimated value of hydroxymethylation, STD = Standard deviation, se= standard error, Min= minimum value of H, and Max= maximum value of H, D= difference, p_adj = p-value adjusted by FDR (Benjamini–Hochberg)

**Table 3:** Statistical analysis using the t-test for the comparison of transformed hydroxymethylation values between animals in the control and treatment groups

Gene	Animals	H	STD	Se	Min	Max	D	*p*-value	*p*-adj (FDR)
*CYP1A1*	C(10)	1.2010	0.4400	0.0142	1.13	1.27	0.0254	0.2371	0.2716
	T(9)	1.1755	0.0463	0.0150	1.10	1.23			
*CYP21A2*	C(10)	1.1280	0.0458	0.0110	1.06	1.19	0.04022	0.0231	0.0693
	T(9)	1.0877	0.0156	0.0116	1.06	1.11			
*CYP1B1*	C(10)	1.1210	0.0369	0.0096	1.05	1.17	0.0221	0.1326	0.1989
	T(9)	1.0988	0.0208	0.0101	1.07	1.13			

where C (10) = control group (non-contaminated feed; *n* = 10 animals); T (9) = ZEN-treated group (*n* = 9 animals), H = estimated value of hydroxymethylation, STD = Standard deviation, se= standard error, Min= minimum value of H, and Max= maximum value of H, D= difference, p_adj = p-value adjusted by FDR (Benjamini–Hochberg).

## Discussion

Given that the CpG islands analyzed were located within gene regions, the observed methylation patterns may be relevant for gene regulation. The literature suggests a correlation between CpG island methylation in both the promoter and exonic regions, which impacts gene expression regulation (Brenet et al. 2011; Hisano et al. 2003; Li et al. 2018). Additionally, as reviewed by Shayevitch (2018), methylation in exonic regions may also be associated with alternative splicing, thereby influencing transcript diversity from a single gene.

In a previous study conducted on the same animals used in this experiment, no significant differences were observed in dry matter intake, digestibility, or performance of heifers (Pião et al. 2023). However, significant alterations were reported in reproductive parameters, particularly oocyte quality (Silva et al. 2021). In the present study, both significant and non-significant epigenetic changes were detected, which may be partially explained by the extensive degradation of ZEN by ruminal microbiota, potentially limiting systemic exposure and contributing to gene-specific epigenetic responses. This microbiota is responsible for degrading approximately 90 to 100% of ingested ZEN (Upadhaya et al. 2010).

Cytochrome P450 enzymes play a central role in hepatic xenobiotic metabolism; therefore, epigenetic modulation of CYP genes may affect the efficiency of ZEN detoxification. Previous studies have shown that *CYP1A1* and *CYP1B1* are expressed in the liver and participate in the metabolism of xenobiotics, including mycotoxins (Guruge et al. 2009; Li et al. 2017; Zepnik et al. 2001). Therefore, epigenetic regulation of these genes may influence hepatic responses to ZEN exposure.

Although *CYP1A1* is associated with detoxification processes, Mróz et al. (2022) observed a reduction in its expression in gilts that ingested ZEN, suggesting a possible suppressive effect on *CYP1A1*. In the present study, no changes were observed in the methylation patterns of the analyzed CpG island in this gene region, suggesting that under the evaluated conditions, ZEN did not influence the epigenetic regulation of *CYP1A1* under the experimental conditions evaluated.

*CYP1B1* is also involved in the endogenous metabolism of steroid hormones (Li et al. 2017). Among these hormones, estradiol is the primary sex hormone in female mammals. According to Hayes et al. (1996), *CYP1B1* plays a key role in its metabolic degradation pathway. The molecular structure of this mycotoxin is similar to that of mammalian 17β-estradiol, leading to hyperoestrogenic syndromes (Zinedine et al. 2007). In the present study, increased DNA methylation in the CpG island of *CYP1B1* was observed in animals exposed to ZEN-contaminated feed, and this effect remained statistically significant after FDR correction, reinforcing the robustness of this finding. Given that the analyzed CpG island is located within the gene body, these methylation changes may be associated with altered transcript processing, including potential effects on alternative splicing.

Moreover, the increased methylation of CYP1B1 in animals exposed to ZEN may contribute to impaired estradiol metabolism and accumulation of estrogenic activity, possibly influencing reproductive efficiency. This hypothesis aligns with previous findings in these same animals, where Silva et al. (2021) observed reduced oocyte quality after ZEN exposure. Therefore, the epigenetic modulation of CYP1B1 may represent a mechanistic link between ZEN ingestion and reproductive impairments in cattle.

The *CYP21A2* gene encodes cytochrome P450 21-hydroxylase, an enzyme involved in steroid hormone biosynthesis, particularly within glucocorticoid and mineralocorticoid pathways (Forest 2004; Mizrachi et al. 2011). This enzyme plays a central role in the metabolism of C21 steroid hormones (Grinberg et al. 2000). Previous gene expression studies in human cell cultures exposed to the mycotoxin alternariol (AOH) have demonstrated upregulation of *CYP21A2* (Frizzell et al. 2013; Kalayou et al. 2014). Although AOH and ZEN are distinct compounds, both exhibit estrogenic properties, which may account for similar regulatory effects on steroidogenic pathways (Demaegdt et al. 2016; Freire et al. 2007; Stypuła-Trębas et al. 2017; Yang et al. 2018; Rogowska et al. 2019).

In the present study, no significant effect on DNA methylation of *CYP21A2* remained after FDR correction; however, a trend toward altered hydroxymethylation was observed. While this finding should be interpreted with caution, it may indicate biologically relevant modulation of steroidogenic pathways, especially considering the well-established endocrine-disrupting effects of ZEN.

## Conclusion

This study demonstrated that ingestion of zearalenone (ZEN)-contaminated feed induced gene-specific epigenetic alterations in Nellore heifers. Increased DNA methylation of *CYP1B1* remained statistically significant after FDR correction, indicating robust epigenetic modulation potentially associated with xenobiotic metabolism. In contrast, the trend toward altered hydroxymethylation observed for *CYP21A2* should be interpreted as suggestive rather than definitive evidence of epigenetic regulation, possibly affecting steroid hormone biosynthesis pathways. No epigenetic changes were detected in *CYP1A1*, reinforcing the gene-specific nature of the response to ZEN exposure. These findings highlight the importance of monitoring ZEN contamination in animal feed and emphasize the need for further studies integrating epigenetic, transcriptomic, and endocrine approaches to clarify its implications for cattle physiology and productivity.

## Data Availability

The data used in this study are available upon request. The authors are committed to providing full access to the relevant data for interested researchers to promote transparency and reproducibility of the results.

## Declarations

### Use of artificial intelligence

The authors declare the assistance of artificial intelligence tools for the grammatical review of this manuscript.

### Use of animals in research

The experimental procedures were approved by the “Animal Care and Use Committee” from the Universidade Federal de Rural do Rio de Janeiro (Institute of Animal Science: #0028-10-2018).
